# Supplementary material for: A novel independence test for somatic alterations in cancer shows that biology drives mutual exclusivity but chance explains most co-occurrence
Source: Genome Biol. 2016 Dec 16;17:261. doi: 10.1186/s13059-016-1114-x (PMC5162102; doi:10.1186/s13059-016-1114-x)
Supplement: Additional file 1 — Supplementary methods and Figures S1–S6. (PDF 897 kb) [file 13059_2016_1114_MOESM1_ESM.pdf]

Supplementary material for *A novel independence test for somatic alterations in cancer shows that biology drives mutual exclusivity but chance explains most co-occurrence*

Sander Canisius[1], John W.M. Martens[2], and Lodewyk F.A. Wessels[1,3]

[1]*Department of Molecular Carcinogenesis, The Netherlands Cancer Institute, Amsterdam, The Netherlands*
[2]*Department of Medical Oncology, Erasmus University Medical Center, Rotterdam, The Netherlands*
[3]*Faculty of EEMCS, Delft University of Technology, Delft, The Netherlands*

Contents

1 Parameter estimation **1**

2 Stratification in pan-cancer analyses **3**

3 False discovery rate control for discrete tests **3**

4 Supplementary figures **4**

1 Parameter estimation

To apply the DISCOVER test, we need estimates of the alteration probabilities p_{ij} for all genes i and all tumors j. These estimates are used as parameters of the Poisson-Binomial distribution used by the test. Here, we show that each alteration probability can be written in terms of two parameters.

$$p_{ij} = \frac{1}{1 + e^{\mu_i + \lambda_j}}$$

For estimating the parameters of the Poisson-Binomial distribution we maximize the information entropy (or equivalently below, minimize the negative of the entropy), subject to the constraints that the expected row and column marginals match the observed ones.

$$\min_p \quad \sum_{i=1}^{n}\sum_{j=1}^{m} p_{ij}\log p_{ij} + (1-p_{ij})\log(1-p_{ij})$$

s.t.

$$\sum_{j=1}^{m} p_{ij} = R_i\ , \qquad 1 \leq i \leq n$$

$$\sum_{i=1}^{n} p_{ij} = C_j\ , \qquad 1 \leq j \leq m$$

$$0 \leq p_{ij} \leq 1\ , \qquad 1 \leq i \leq n, 1 \leq j \leq m$$

where

$$R_i = \sum_{j=1}^{m} x_{ij}$$

$$C_j = \sum_{i=1}^{n} x_{ij}$$

We first turn this constrained optimization problem into an unconstrained one by defining the Lagrangian dual. The Lagrangian is as follows.

$$\begin{aligned}\mathcal{L}(p,\mu,\lambda) = \sum_{i,j} p_{ij}\log p_{ij} + (1-p_{ij})\log(1-p_{ij}) + \sum_i \mu_i(\sum_j p_{ij} - R_i) \\ + \sum_j \lambda_j(\sum_i p_{ij} - C_j)\end{aligned}$$

which we rewrite to

$$\begin{aligned}\mathcal{L}(p,\mu,\lambda) = \sum_{i,j} p_{ij}\log p_{ij} + (1-p_{ij})\log(1-p_{ij}) + \sum_i \mu_i \sum_j p_{ij} - \sum_i \mu_i R_i \\ + \sum_j \lambda_j \sum_i p_{ij} - \sum_j \lambda_j C_j\end{aligned}$$

We then optimize the Lagrangian with respect to the variables p_{ij} by setting the partial derivatives to 0 and solving for those variables.

$$\frac{\partial \mathcal{L}}{\partial p_{ij}} = \log p_{ij} - \log(1-p_{ij}) + \mu_i + \lambda_j = 0$$

From this, we derive that

$$\log \frac{p_{ij}}{1-p_{ij}} = -\mu_i - \lambda_j$$

The left-hand side of this equation is the familiar logit function, the inverse of which is the logistic function. Hence, we obtain the following expression for p_{ij}.

$$p_{ij} = \frac{1}{1 + e^{\mu_i + \lambda_j}}$$

With this, we can formulate the Lagrangian dual as follows.

$$\max_{\mu,\lambda} \sum_{i,j} p_{ij} \log p_{ij} + (1 - p_{ij}) \log(1 - p_{ij}) + \sum_i \mu_i (\sum_j p_{ij} - R_i) + \sum_j \lambda_j (\sum_i p_{ij} - C_j)$$

where p_{ij} is defined as above.

2 Stratification in pan-cancer analyses

Mutual exclusivity of somatic alterations is often interpreted as a reflection of the functional redundancy of those alterations. This view is supported by the mutual exclusivity frequently found within cancer-driving pathways. When performing a pooled analysis of multiple cancer types however, another, more trivial kind of mutual exclusivity may be identified. Such mutual exclusivities arise simply because the mutually exclusive alterations are specific to a cancer type. If not properly adjusted for, they may even dominate the results, making it more difficult to identify the functionally interesting findings.

To illustrate this, we pooled TCGA's colon adenocarcinoma (COAD) and breast cancer (BRCA) mutation data and tested for mutual exclusivity between mutations in *KRAS* and *CDH1*. In the pooled data, *KRAS* is mutated in 65 tumors, *CDH1* in 114 tumors, but both are mutated in only 3 tumors. If we do not adjust for cancer type, DISCOVER estimates the expected number of tumors with both genes mutated to be 21. As such, there is every reason to call this mutually exclusive, and indeed the test is highly significant ($P = 1.5 \times 10^{-8}$). However, realizing that 59 of the *KRAS* mutations are in the colon tumors, and 109 of the *CDH1* mutations are in the breast tumors, it becomes clear that the mutual exclusivity is almost perfectly confounded by cancer type. Stratification of the DISCOVER test corrects for this type of confounding: the expected number of tumors with mutations in both genes is estimated to be 6, and the test for mutual exclusivity is not significant ($P = 0.12$).

3 False discovery rate control for discrete tests

The Benjamini-Hochberg procedure is a popular procedure to correct for multiple testing in genomics analyses. It controls the false discovery rate (FDR) at a specified level. To do so, it uses the following simple method to estimate the expected FDR for a given significance level α. In an analysis where N tests are performed, by definition αN tests are expected to yield a P-value below α by chance. If in reality, m tests are significant at level α, then the expected FDR is $\frac{\alpha N}{m}$. The assumption that makes this work is that under the null hypothesis the tests' P-values are distributed uniformly and continuously. Based on this assumption, αN is a valid estimate of the expected number of false discoveries.

In the case of statistical tests with a discrete test statistic, such as DISCOVER, the null distribution tends not to be truly uniform, but shows a skew towards one instead. Moreover, due to differences in parameters, the particular null distribution for each individual test often differs as well. In the case of such discrete test statistics, the Benjamini-Hochberg procedure still controls the FDR, but it does so in a highly conservative way. Due to the null distribution's skew towards one, αN is likely

to be an overestimate of the actual number of false discoveries. As a consequence, the FDR for a significance level α is estimated too high.

The adaptation of the Benjamini-Hochberg procedure for discrete test statistics that we used [1] takes advantage of the fact that for discrete null distributions it is possible to enumerate the entire distribution. As continuous distributions cover infinitely many values—and thus cannot be enumerated—the assumption of uniformity is necessary to estimate that under the null hypothesis the proportion of P-values below α is itself equal to α. For discrete tests—where it is possible to enumerate the actual null distribution—we can determine the true proportion of P-values below α and use this as a more accurate estimate of the number of false discoveries. For the exact details of the procedure, we refer to Carlson et al. [1].

References

[1] Carlson, J.M., Heckerman, D., Shani, G.: Estimating false discovery rates for contingency tables. Technical Report MSR-TR-2009-53 (2009). `http://research.microsoft.com/apps/pubs/default.aspx?id=80571`

4 Supplementary figures

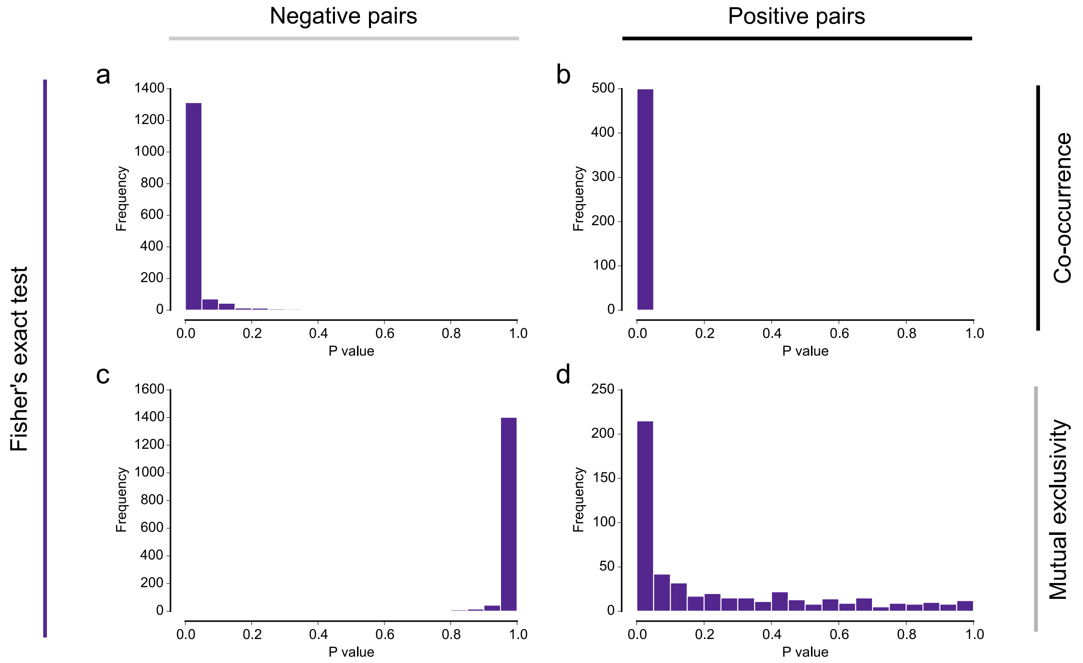

Figure S1: Histograms of P-values obtained on simulated data using Fisher's exact test. The P-values apply to gene pairs with three different types of relation: gene pairs with independent alterations (a, c), gene pairs with co-occurring alterations (b), and gene pairs with mutually exclusive alterations (d).

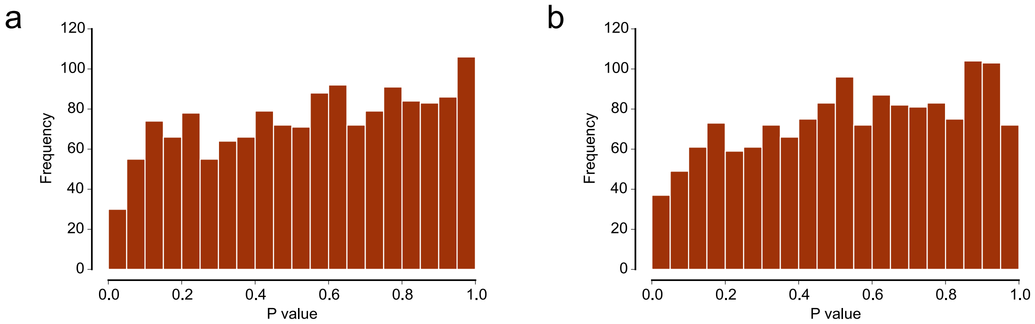

Figure S2: Histograms of P-values obtained by testing independent gene pairs for either co-occurrence (a) or mutual exclusivity (b) using the Binomial test. Simulated alteration data were generated in such a way that gene alteration frequencies resemble those in real tumors. Alteration frequencies of tumors are similar for all tumors.

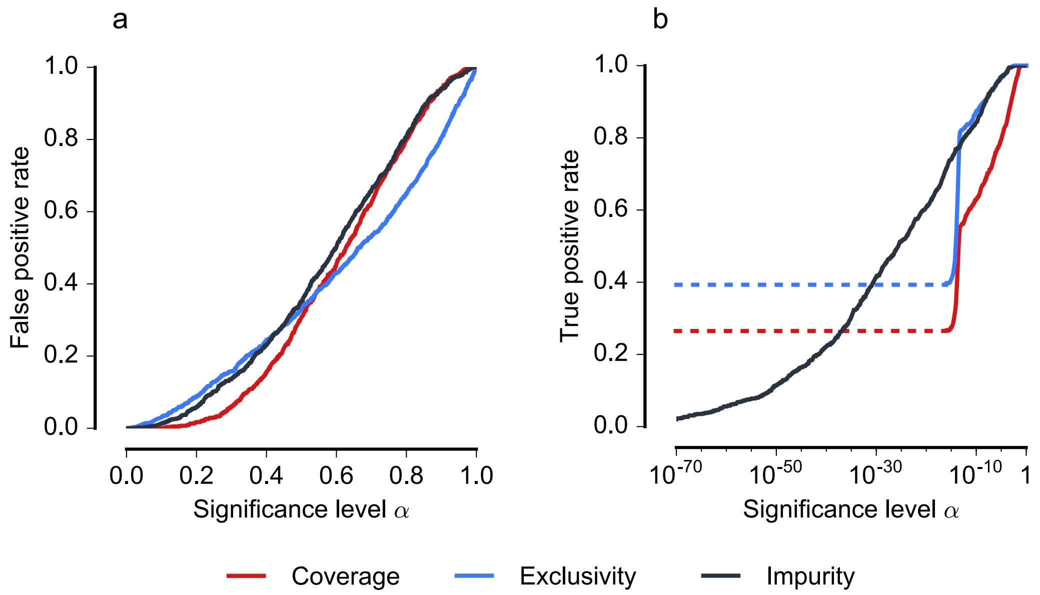

Figure S3: Performance on simulated gene sets of the DISCOVER test based on three alternative statistics: Coverage, the number of tumors that have an alteration in at least one of the genes; Exclusivity, the number of tumors that have an alteration in exactly one gene; Impurity, the number of tumors that have an alteration in more than one gene. (a) P-value reliability curves. The false positive rate should not exceed the significance level α. In such a case, the calibration curve will be below the diagonal. This is the case for all three statistics. (b) Sensitivity curves: more sensitive tests will attain higher true positive rates at lower significance levels. The dotted lines mark a range of significance levels where all lower P-values are 0.

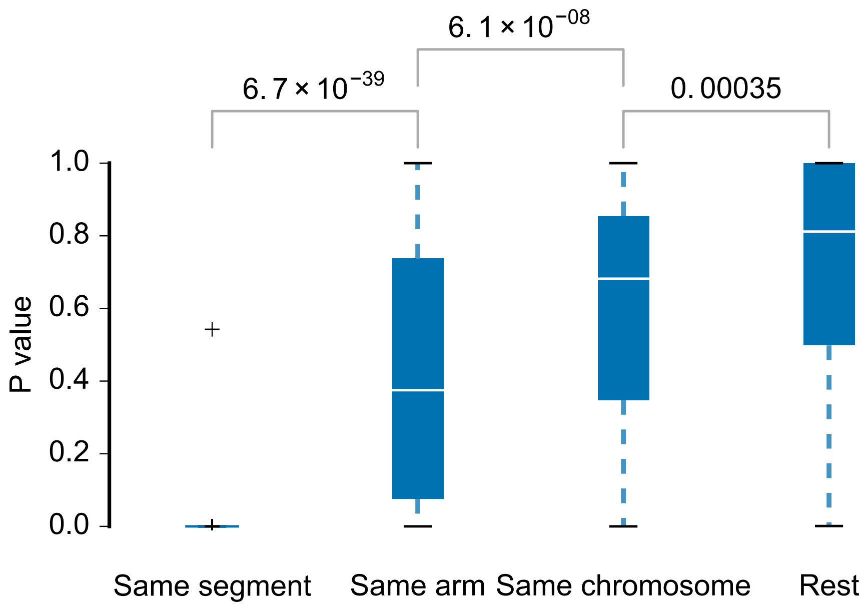

Figure S4: Comparison of P-values obtained when testing for co-occurrence between genes within the same recurrently altered segment, within the same chromosome arm, within the same chromosome, and across chromosomes. Differences in mean are tested with the Mann-Whitney U test.

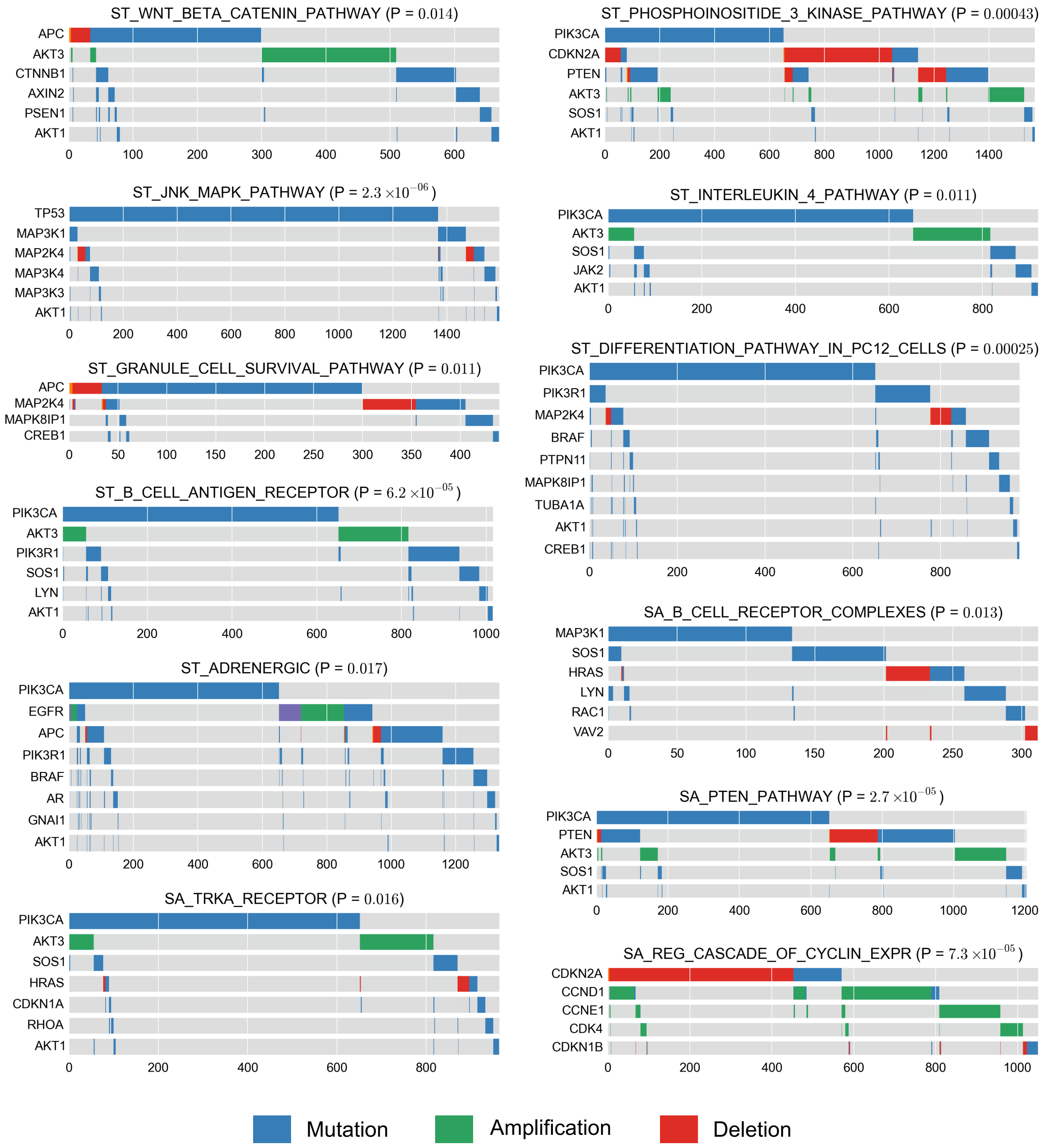

Figure S5: Overview of the significantly mutually exclusive gene sets from the MSigDb canonical pathway collection.

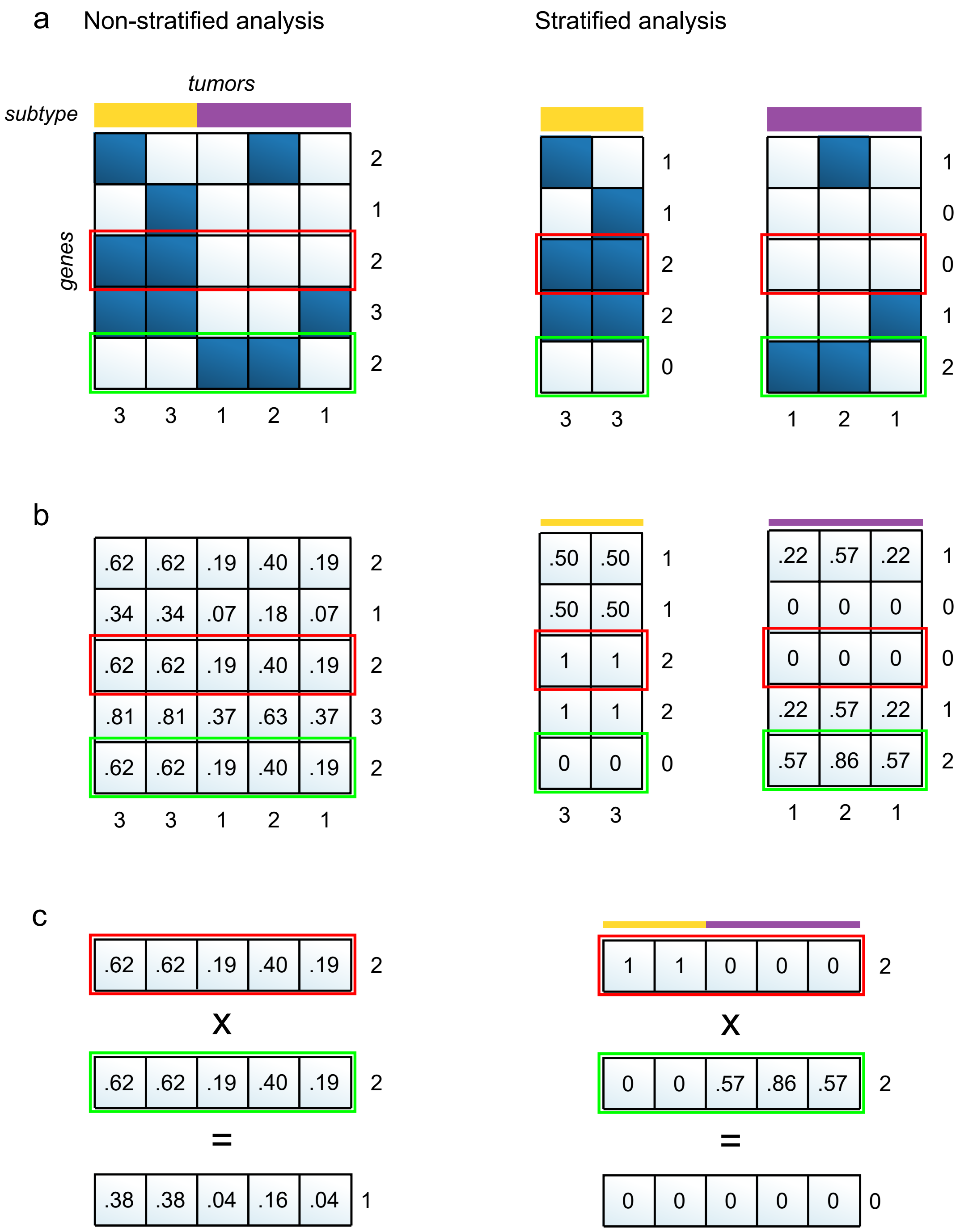

Figure S6: Illustration of stratification in DISCOVER. (a) Alteration matrix for tumors of two different cancer (sub)types. These subtypes are marked by yellow and purple. The two genes highlighted in red and green are never co-altered in the same tumor. Moreover, alterations in either one of the two genes are exclusive to one of the two subtypes. (b) Estimation of the alteration probabilities. In the non-stratified setting (left), estimation proceeds as explained previously. The expected numbers of

alterations per gene and per tumor match the observed numbers. In the stratified setting (right), the original alteration matrix $\mathcal{X}$ is split into individual alteration matrices $\mathcal{X}_1$ and $\mathcal{X}_2$ for each subtype. The alteration probability matrices $\mathcal{P}_1$ and $\mathcal{P}_2$ are then estimated for each matrix separately. As a result, the expected numbers of alterations per gene not only match the observed numbers overall, but also within each subtype. For the subsequent step, the subtype-specific probability matrices are concatenated and treated as a single probability matrix $\mathcal{P} = [\mathcal{P}_1\ \mathcal{P}_2]$. (c) Estimation of the expected number of tumors with alterations in both genes. Using the non-stratified alteration probabilities (left), the number of co-altered tumors expected by chance equals 1. Using the stratified alteration probabilities (right), this number equals 0. This correctly recognizes that within a subtype, only one of the two genes is ever altered. Hence, the observed exclusivity of alterations in the two genes is fully explained by the tumors' subtype. When taking into account subtype, the observed number of co-altered tumors is not lower than expected by chance.
